# Comprehensive subspecies identification of 175 nontuberculous mycobacteria species based on 7547 genomic profiles

**DOI:** 10.1080/22221751.2019.1637702

**Published:** 2019-07-09

**Authors:** Yuki Matsumoto, Takeshi Kinjo, Daisuke Motooka, Daijiro Nabeya, Nicolas Jung, Kohei Uechi, Toshihiro Horii, Tetsuya Iida, Jiro Fujita, Shota Nakamura

**Affiliations:** a Department of Infection Metagenomics, Genome Information Research Center, Research Institute for Microbial Diseases, Osaka University, Suita, Japan; b Department of Infectious, Respiratory, and Digestive Medicine, Graduate School of Medicine, University of the Ryukyus, Nakagami-gun, Japan; c Laboratory of Pathogen Detection and Identification, International Research Center for Infectious Diseases, Research Institute for Microbial Diseases, Osaka University, Suita, Japan; d Division of Clinical Laboratory and Blood Transfusion, University of the Ryukyus Hospital, Nakagami-gun, Japan; e Integrated Frontier Research for Medical Science Division, Institute for Open and Transdisciplinary Research Initiatives, Osaka University, Suita, Japan

**Keywords:** Nontuberculous mycobacteria, multi-locus sequence typing, next-generation sequencing, comparative genomics, pulmonary diseases

## Abstract

The prevalence of nontuberculous mycobacteria (NTM) pulmonary diseases has been increasing worldwide. NTM consist of approximately 200 species and distinguishing between them at the subspecies level is critical to treatment. In this study, we sequenced 63 NTM genomes, 27 of which were newly determined, by hybrid assembly using sequencers from Illumina and Oxford Nanopore Technologies (ONT). This analysis expanded the available genomic data to 175 NTM species and redefined their subgenus classification. We also developed a novel multi-locus sequence typing (MLST) database based on 184 genes from 7547 assemblies and an identification software, mlstverse, which can also be used for detecting other bacteria given a suitable MLST database. This method showed the highest sensitivity and specificity amongst conventional methods and demonstrated the capacity for rapid detection of NTM, 10 min of sequencing of the ONT MinION being sufficient. Application of this methodology could improve disease epidemiology and increase the cure rates of NTM diseases.

## Introduction

The *Mycobacterium* genus belongs to the Actinobacteria phylum and includes three major human pathogens: *Mycobacterium tuberculosis* (MTB), *Mycobacterium leprae*, and nontuberculous mycobacteria (NTM) species. Unlike MTB and *M. leprae*, NTM species are opportunistic pathogens existing in common environmental sources such as water and soil [1,2]. Recently, the prevalence of NTM pulmonary disease (NTM-PD) has been increasing in both developed and developing countries [3–8]. It has also been reported that race, ethnicity, sex, and geographical factors affect the predominant species [4,8]. Thus, NTM diseases are now recognized as a new global health concern.

Approximately 200 NTM species have been identified, of which approximately 140 are pathogenic to humans and animals, and the remaining 60 species are also potentially pathogenic [9–11]. Of these, the *Mycobacterium avium* complex (MAC: *M. avium*, *Mycobacterium intracellulare*, and others), *Mycobacterium kansasii*, and the *Mycobacteroides abscessus* complex (*M. abscessus* subsp. *abscessus*, *M. abscessus* subsp. *massiliense*, and *M. abscessus* subsp. *bolletii*) are the major causative agents of NTM-PD [7,12]. These two complexes are further classified into many subspecies that show different susceptibilities to chemotherapies [13–15]. This diversity of NTM subspecies represents a significant obstacle against successful treatment, and current therapies require prolonged treatment and often lead to insufficient clinical outcomes due to the lack of subspecies identification [16]. Therefore, identifying NTM at a subspecies level is an essential step prior to treatment [14,15].

Conventional culture and acid-fast staining methods are still crucial for laboratory-based NTM diagnosis. Mass spectrometry (MS) analysis is starting to gain popularity in large hospitals [17,18]. Most current methods for definitive species identification are based on molecular assays, such as nucleic acid hybridization and DNA sequencing of one or several house-keeping genes [19,20] (i.e. multi-locus sequence typing [MLST]). Several methods based on genomic information achieve subspecies-level resolution using ribosomal genes, and core and whole genomic information [21–23]. Despite the growing demand for the genomic information to identify NTM at the subspecies level, available public databases are lacking in terms of quality and comprehensiveness. Recent comprehensive genomic studies have added genomic data for several NTM species [9,24], however, available genomic data are still limited to the assemblies of 148 species as of December 1, 2017, in NCBI RefSeq and GenBank databases.

In this work, we expanded the NTM genomic data by 63 species, of which 27 species were newly determined and the remaining 36 species were re-sequenced by Illumina and Oxford Nanopore Technologies (ONT). By combining these sequenced genomes and the currently available genomic data, we constructed a novel MLST database of 175 NTM species and developed an identification method (designated as mlstverse) that enables comprehensive detection of NTM at the subspecies level.

## Materials and methods

### Cultivation of NTM strains and DNA extraction

The 63 NTM strains sequenced in this study were provided by RIKEN BRC (BioResource Research Center, Saitama, Japan). The selection criteria were as follows: they had a binomial name and either a genome not yet sequenced or assemblies comprising contigs <1 Mb. Species with no valid binomial name (e.g. *Mycobacterium* sp.) were excluded. These 63 strains described in Table S1 were cultivated on solid Middlebrook 7H10 agar with OADC Enrichment, which was prepared by diluting the following reagents to 1.0 L with distilled water (pH 7.2): 19 g Middlebrook 7H10 agar (Becton, Dickinson and Company [BD] Difco, Franklin Lakes, NJ, USA), 5.0 ml glycerol, 895 ml distilled water, 100 ml Middlebrook OADC Enrichment (BD BBL), R agar (10.0 g bacto peptone, BD Difco), 5.0 g yeast extract (BD Difco), 5.0 g malt extract (BD Difco), 5.0 g casamino acids (BD Difco), 2.0 g beef extract (BD Difco), 2.0 g glycerol, 50.0 mg Tween 80, 1.0 g MgSO_4_·7H_2_O, and 15.0 g. The plates were incubated at 25, 30, 37, or 45 °C in an incubation oven PIC-100 (As ONE Corporation, Osaka, Japan) for 3 days to 7 weeks. Genomic DNA was extracted from bacterial colonies using the DNeasy PowerSoil Kit (Qiagen, Hilden, Germany).

### Library preparation and sequencing with an Illumina HiSeq2500 instrument

Genomic DNAs were sheared to 600 bp using a focused-ultrasonicator S220 (Covaris, Inc., Woburn, MA, USA) and snap-cap microtubes (Covaris) with a peak incident power of 105 W, a duty factor of 5%, 200 cycles per burst, and a 70 s treatment time. A genomic DNA library was prepared from 1 µg sheared DNA as we described previously [25], based on ligation-based Illumina multiplex library preparation (LIMprep) with HiFi DNA polymerase (Roche, Basel, Switzerland). Sequences were obtained as dual-indexed, 100 bp paired-end reads using HiSeq SBS V4 (200 Cycles) and an Illumina HiSeq2500 instrument.

### Library preparation and sequencing using ONT MinION

Genomic DNA (1.5–2.5 μg) was sheared in g-TUBEs (Covaris) and centrifuged at 7200 rpm (4868 x g) for 60 s in Eppendorf 5424 centrifuges with rotor FA-45-24-11. One microgram sheared DNA was used for each library preparation and prepared with the Ligation Sequencing Kit (ONT, Oxford, UK) and the Native Barcoding Kit (ONT), according to the manufacturers’ instructions. Sequencing runs were performed using a MinION instrument (ONT).

### Assembly and annotation

The genome assembly was performed with Canu [26] (v. 1.7) using long reads obtained from MinION after filtering reads less than 4000 bp long. Contigs were polished with bwa [27] (0.7.17-r1188) and pilon [28] (v. 1.22) using the short reads obtained from the HiSeq2500 instrument. Circular genomes with the “*suggestCircular*” flag in their sequence headers were trimmed at both ends of the sequences and rotated to set the start of the sequence to the origin of replication using Circlator. The polished genomes were then put through the RAST annotation pipeline [29].

### Cultivation of clinical samples and MS analysis

The NTM strains were isolated from clinical specimens of patients who visited or admitted to the University of the Ryukyus Hospital between May 2015 and August 2016, and stored in skim milk at −80 °C. These clinical isolates were inoculated on 2% Ogawa medium, and the cultures were incubated at 35 °C. A 1-µL loopful of colonies was picked and transferred into a tube with 0.6 mm zirconia/silica beads and 70% ethanol, followed by vortexing for 15 min and incubation at room temperature (approximately 25°C) for another 10 min. After centrifugation, the pellets were uniformly dispersed by successive washing with 70% formic acid and acetonitrile. After centrifugation, 1 µL of each supernatant was transferred to the target slide. Once the sample was dry, 1 µL of CHCA matrix (bioMérieux SA, Marcy L’Eoile, France) was applied to each slide and allowed to dry prior to analysis. The identifications were run in automatic mode of Vitek MS v3.0 system (bioMérieux SA). For each isolate, a single extraction was performed and spotted in quadruplicate. For each run, *Escherichia coli* (ATCC 8739; American Type Culture Collection, Manassas, VA, USA) was used for calibration and quality-control testing, according to the manufacturer’s protocols.

### Comparative analysis

Assemblies of NTM species were collected from NCBI RefSeq and GenBank, as well as our newly sequenced genomes. We collected high-quality assemblies with long N50 sequences in the reference genome, representative genomes, or normal assemblies. Homology searching among the species was performed for each coding sequence (CDS) obtained from RAST using blastn [30] (blast 2.6.0+). CDSs clustered with a percent identity of amino acid sequence of ≥80% and a length ratio of ≤0.8 were clustered as orthologs. The length ratio was calculated by dividing the length of the shorter sequence by the length of the longer one. Orthologs uniquely conserved in 80% or more of the 175 NTM species were extracted and defined as core genes. After aligning core genes individually using MAFFT [31] (v. 7.313), core genomes were constructed by merging sequences of core genes in the same order. For sequence alignments, the missing genes were denoted by consecutive gap symbols (-). A phylogenetic tree was created by Ward’s method with pairwise distance, using an identity matrix for the distance and core genomes. For core genes conserved in 100% of the NTM species, construction of the core genome and phylogenetic tree were performed in the same way.

### 16S rRNA gene sequence analysis

The 16S rRNA gene was amplified from genomic DNA by PCR using the primers 27F (5’-AGAGTTTGATCMTGGCTCAG-3’), 1492R (5’-TACGGYTACCTTGTTACGACTT-3’), 337F (5’-GACTCCTACGGGAGGCWGCAG-3’), and 1391R (5’-GACGGGCGGTGTGTRCA-3’). PCR was performed with 25 ng of genomic DNA template using 2X KAPA HiFi HotStart ReadyMix (Roche) and 0.3 μM each primer in a LifeECO thermal cycler (Bioer Technology, Hangzhou, China), with the following thermocycling programme: denaturation at 95°C for 3 min; 30 cycles of 98°C for 20 s, 60°C for 15 s, and 72°C for 30 s; and a final elongation at 72°C for 1 min. The 16S rRNA gene sequences were determined with a 3130xI Genetic Analyzer (Applied Biosystems, Foster, CA, USA) using amplified fragments and PCR primers. Partial rRNA sequences of approximately 1.5 kb were compared to the reference sequences of the *Mycobacterium* genus in the SILVA database, using blastn. Sequences showing ≥98.7% identity were selected.

### Library preparation and sequencing clinical isolates

DNA shearing was performed as described above in the section entitled, “Library preparation and sequencing with an Illumina HiSeq2500 instrument.” We prepared a library from 1 μg sheared DNAs using KAPA Library Preparation Kits (Roche) by ligation-based Illumina multiplex library preparation (LIMprep). The sequencing run was performed using the MiSeq Reagent Kit v3 (600 Cycles, Illumina).

### Identification using MLST-based methods

The software mlsverse uses a BAM format file as an input file. Any mapping software, such as bwa [27], bowtie [32], and minimap2 [33], can be used for mapping. We used bwa mem for Illumina data and minimap2 for ONT data. The reference database bundled with the mlstverse software includes sequence data of each locus for mapping. The indexing of the BAM file was performed using samtools [34]. We performed mlstverse using default parameters (min_ratio = 0.1; th.score = 0.1; and method = “default”). The software metamlst [22] uses a FASTQ file as input. We executed metamlst using default parameters and the database metamlstDB_2017.db. The software pubmlst [23] requires an assembled sequence file, for which we used the software SPAdes [35] for assembly. The assembled FASTA file was used as input for the pubmlst web interface.

## Results

### Genome sequencing of 63 NTM species

**Figure 1.**
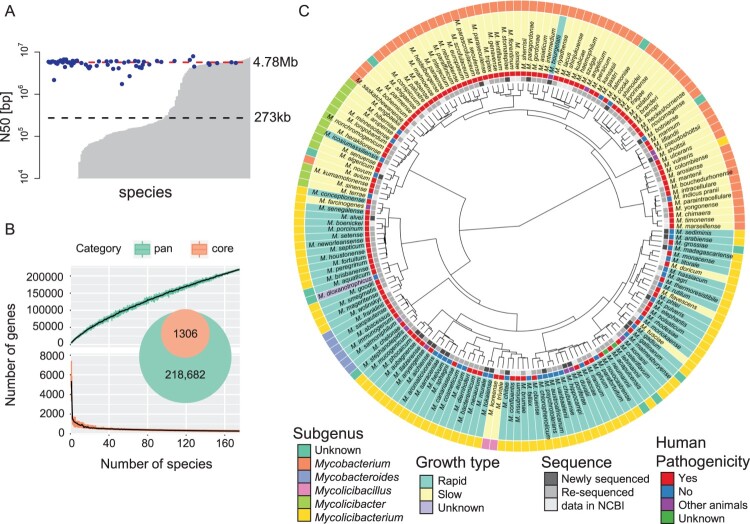
**Taxonomic analysis using all *Mycobacterium* species.** A) Assembly quality. The bar plot indicates the N50 length in each species. The grey bar was available with the NCBI assemblies. The x-axis is sorted in ascending order with y-axis value. The blue dots show assemblies obtained in this work. The black and red dashed lines show the median N50s calculated using assemblies available from NCBI and our assemblies (also see Table S1). Growth type was defined according to the time required to grow bacterial colonies [i.e. rapid (3–7 days) and slow (>7 days)]. B) Core and pan genome analysis. Gene clusters were discriminated based on differences in the percent identity and length. The numbers of core (bottom) and pan (top) genes are shown, based on the number of NTM species. The error bars were calculated from 1000 replicates of randomly selected species. C) Comparative analysis. A phylogenetic tree was constructed using the 80% core genome (also see Figure S1). Filled colours correspond to the subgenus, growth type, pathogenicity, and assembly availability.

Genome sequences for 148 out of approximately 200 known NTM species are currently available in the NCBI RefSeq and GenBank databases. However, the quality of those genomic sequences is lacking, as the assemblies with an N50 exceeding 1 Mb account for only 66 species. To complement the genomic information for NTM, we performed genome sequencing on 63 NTM species, including 27 newly sequenced species and 36 re-sequenced species (Table S1). We applied a hybrid assembly method to construct their respective genomes using next-generation sequencing (NGS) data from Illumina and ONT. The new dataset covered 175 species and reached a median N50 of 4.78 Mb, which was better than the previous median of 273 kb. (Figure 1A).

### Core genome and pan genome of 175 NTM species

Using the improved NTM genome data, we investigated phylogenetic relationships among 175 species. To define the core and pan genomes of those species, the orthologs of each gene were clustered by homology searching. We found that the 288 genes were 100% conserved in these 175 species (100% core genome) and that 1,306 genes were 80% conserved (80% core genome). The pan genome of these species constituted 218,682 genes (Figure 1B). The phylogenetic tree constructed using the 80% core genome strongly reflected differences in the growth type, but not in pathogenicity to humans or animals (Figure 1C). The phylogenetic tree constructed with the 100% core genome gene set showed a similar tendency (Figure S1).

In the constructed tree in Figure 1C, the newly sequenced 27 species (excluding one *M. bourgelatii* species) were classified into a consistent growth type. We identified the following subgenera among these species: the subgenus *Mycobacterium* (*M. tuberculosis-simiae* clade) for *M. botniense*, *M. talmoniae*, and *M. shigaense*; the subgenus *Mycolicibacterium* (*M. fortuitum* clade) for *M. aquaticum*, *M. dioxanotrophicus*, *M. neumannii*, *M. lehmannii*, *M. gallinarum*, and *M. grossiae*; and the subgenus *Mycobacteroides* (*M. chelonae*　clade) for *M. stephanolepidis*. Previously, *M. avium* was classified as belonging to the *M. terrae* clade [10]. However, our results suggested that it belongs to the closely related *M. simiae* clade. *M. bourgelatii* reportedly exhibits rapid growth [36]; however, it was classified into the *Mycobacterium* genus, in which almost all species exhibit slow growth. To verify *M. bourgelatii* classification, we compared its conserved signature indels (CSIs) and conserved signature proteins (CSPs) [10] with those of other species belonging to the *Mycobacterium* genus, revealing that the genetic features of *M. bourgelatii* harbored two of the three CSIs and three of the four CSPs that matched those of the *Mycobacterium* genus (Figure S4). These results enabled us to expand the phylogenetic relationship of 175 NTM species by including the new 27 species with subgenus information.

### Software development for NTM identification

To develop new software for identifying NTM species, we first constructed an MLST database (mlstdb.NTM) using the genomic information from the 175 NTM species described above. We collected 7,547 assemblies of *Mycobacterium* consisting of 7,484 assemblies from NCBI and 63 assemblies from our newly assembled sequences. To distinguish NTM from other *Mycobacterium* species, these genomic profiles included assemblies of not only NTM but also the MTB complex, *M. leprae*, and other species belonging to the *Mycobacterium* genus. We extracted the sequences of 184 genes related to ribosomal proteins, ribosomal RNA genes, several reference genes, antibiotic-resistant genes, and pathogenic genes. The combination of sequences for each gene in an assembly was stored as a sequence type (ST) with assembly information: species, strain, and assembly level.

**Figure 2.**
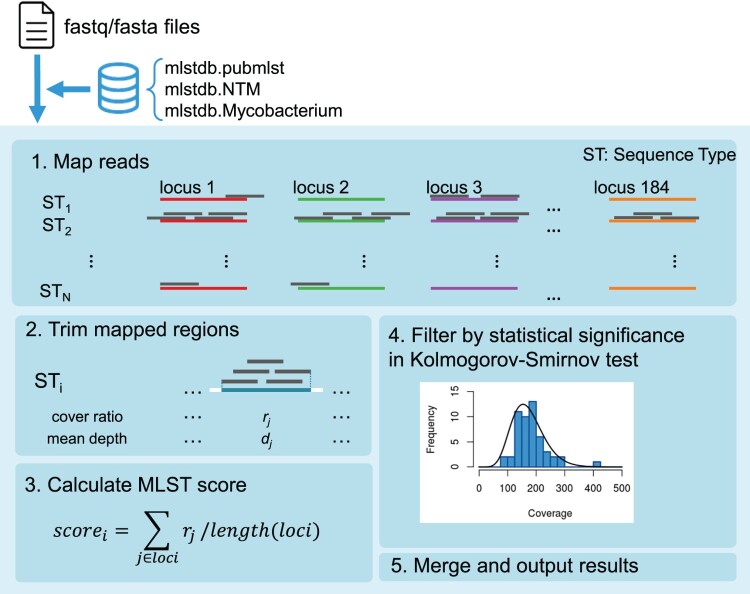
**Pipeline workflow.** Mlstverse uses an fasta or fastq file as input and outputs an identification result as a table, using a given database.

We then developed mlstverse, an identification software package for bacteria based on MLST (Figure 2). As the first step of identification, mlstverse loads the sequences mapped on the database. In the second step, MLST scores are calculated from the mapping ratio and coverage for each gene of all STs in the mlstdb.NTM database using the mapping result (bam format). To verify the identification results, mlstverse also performs a statistical hypothesis test. When enough reads are obtained, the number of reads mapped for each gene should follow a negative binomial distribution. Then, mlstverse checks the distribution of the results using a Kolmogorov–Smirnov test. Finally, mlstverse outputs the detected species and their MLST scores as a list. This software can be universally applied for detecting any given bacterium, using a suitable MLST database.

### Comparing the performance of mlstverse with conventional methods

To test the performance of mlstverse and the constructed database, we examined the detection of 29 *Mycobacterium* strains isolated from clinical samples (Figure S2). Species detection was also performed with other identification methods, such as MS analysis, 16S rRNA gene analysis, two MLST-based methods, and whole-genome shotgun (WGS) sequencing (Table 1). Of these 29, 16 samples were identified at the species level by MS analysis, with one misdetection of *M. yongonense* as *M. intracellulare*, and the remaining 13 samples were not identified.

**Table 1.** Comparison of NTM-identification methods. Analysis with the WGS, MS, 16S rRNA sequence analysis, and MLST-based metaMLST, pubmlst, and mlstverse methods is shown (also see Supplementary Tables 2 and 3).

WGS	16S	VITEK MS	metaMLST (Zolfo et al. 2017)	pubmlst (Jolley & Maiden 2010)	mlstverse (this study)	sample#
*M. abscessus* subsp. *abscessus*	multiple hit	*M. abscessus*	*M. abscessus*	*M. abscessus*	*M. abscessus* subsp. *abscessus*	4
*M. abscessus* subsp. *abscessus*	multiple hit	*M. abscessus*	*M. abscessus*	*M. abscessus*	*M. abscessus* subsp. *abscessus*	5
*M. abscessus* subsp. *massiliense*	multiple hit	*M. abscessus*	*M. massiliense*	*M. abscessus*	*M. abscessus* subsp. *massiliense*	8
*M. avium* subsp. *hominissuis*	multiple hit	*M. avium*	–	*M. avium*	*M. avium* subsp. *hominissuis*	12
*M. avium* subsp. *hominissuis*	multiple hit	*M. avium*	–	*M. avium*	*M. avium* subsp. *hominissuis*	13
*M. chelonae*	multiple hit	*M. chelonae*	–	*M. chelonae*	*M. chelonae*	1
*M. chelonae*	multiple hit	*M. chelonae*	–	*M. chelonae*	*M. chelonae*	6
*M. chelonae*	multiple hit	*M. chelonae*	–	*M. chelonae*	*M. chelonae*	7
*M. chelonae*	multiple hit	*M. chelonae*	–	*M. chelonae*	*M. chelonae*	9
*M. chelonae*	multiple hit	*M. chelonae*	–	*M. chelonae*	*M. chelonae*	10
*M. fortuitum* subsp. *cacetamidolyticum*	multiple hit	*M. fortuitum*	–	*M. fortuitum*	*M. fortuitum* subsp. *acetamidolyticum*	2
*M. yongonense*	multiple hit	*M. intracellulare*	–	*M. yongonense*	*M. yongonense*	3
*M. intracellulare*	multiple hit	*M. intracellulare*	–	*M. intracellulare*	*M. intracellulare*	11
*M. colombiense*	multiple hit	–	–	*M. colombiense*	*M. colombiense*	25
*M. europaeum*	multiple hit	–	–	*M. europaeum*	*M. europaeum*	26
*M. gordonae*	multiple hit	–	–	*M. gordonae*	*M. gordonae*	14
*M. gordonae*	multiple hit	–	–	*M. gordonae*	*M. gordonae*	15
*M. gordonae*	multiple hit	–	–	*M. gordonae*	*M. gordonae*	18
*M. gordonae*	*M. paragordonae*	–	–	*M. gordonae*	*M. gordonae*	22
*M. gordonae*	multiple hit	–	–	*M. gordonae*	*M. gordonae*	23
*M. gordonae*	*M. paragordonae*	–	–	*M. gordonae*	*M. gordonae*	24
*M. gordonae*	*M. paragordonae*	–	–	*M. gordonae*	*M. gordonae*	28
*M. kansasii*	multiple hit	–	–	*M. kansasii*	*M. kansasii*	20
*M. lentiflavum*	multiple hit	–	–	*M. lentiflavum*	*M. lentiflavum*	16
*M. lentiflavum*	multiple hit	–	–	*M. lentiflavum*	*M. lentiflavum*	19
*M. lentiflavum*	*M. lentiflavum*	–	–	*M. lentiflavum*	*M. lentiflavum*	27
*M. lentiflavum*	multiple hit	–	–	*M. lentiflavum*	*M. lentiflavum*	29
*M. wolinskyi*	multiple hit	–	–	*M. wolinskyi*	*M. wolinskyi*	17
*M. wolinskyi*	multiple hit	–	–	*M. wolinskyi*	*M. wolinskyi*	21
Accuracy	0.03 (0/1/29)	0.45 (0/13/29)	0.10 (0/3/29)	1.00 (0/29/29)	1.00 (6/29/29)	

Sequence analysis of 16S rRNA genes is the usual choice for *Mycobacterium* identification. To examine the specificity associated with 16S rRNA gene analysis, we performed full-length 16S PCR using a universal primer set and sequenced the products by Sanger sequencing. We conducted a homology search using a SILVA database, which contained 166 *Mycobacterium* species. Although all strains showed ≥98.7% identity among their 16S rRNA gene sequences, the identity of only one species was determined uniquely and correctly. The remaining 28 strains showed multiple candidates (Table S2). For the strains identified as *M. paragordonae* using other methods, the 16S rRNA gene analysis yielded *M. gordonae* as a result. With the remaining strains, for which multiple candidate species were found, the candidates with the highest homology were consistent with the homology search results of WGS.

We performed two MLST-based identification analyses (metaMLST [22] and pubmlst [23]) along with mlstverse. With the metaMLST results, only three strains were identified, and the candidates did not contain subspecies information. The pubmlst results showed candidates that were consistent with the WGS results for all 29 samples. However, neither method identified the subspecies of *M. abscessus*, *M. avium*, or *M. fortuitum*.

**Figure 3.**
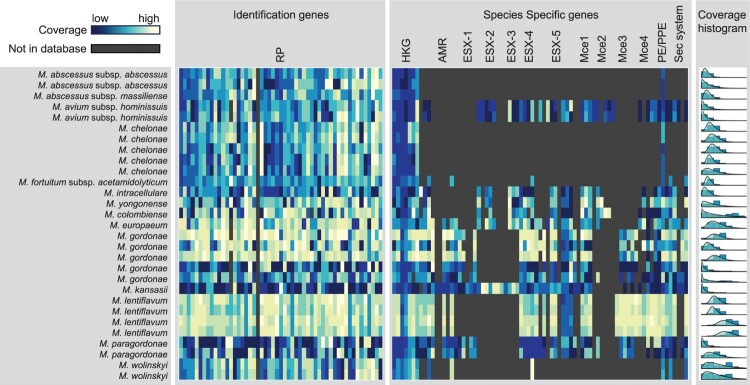
**Distribution of mapped reads in 29 clinical samples.** The coverage of each gene in each sample is shown as a heat map for 53 ribosomal proteins genes and 131 species-specific genes. The coverage distribution is also shown on the right, with a binomial distribution fitting.

We next tested our mlstverse programme. Our method identified all strains including *M. abscessus*, *M. avium*, and *M. fortuitum*, which could not be identified by the other methods (Table 1) at the subspecies level. When scoring was based on the genes possessed by each ST, we obtained an average best score of 0.90 for each of the 29 samples (Table S3). The best scores were separated from the next-best score by 0.50, on average. To visualize what genes were present in each profile, we created a heat map showing the output of mlstverse. For each sample, labelled according to the species, the highest coverage score was displayed for each gene (Figure 3). The 184 genes used for constructing the MLST database are labelled as 53 identification genes and 131 species-specific genes. Although genes related to the ribosomal complex or reference genes were conserved for most *Mycobacterium* species, genes related to antibiotic resistance or pathogenicity differed markedly, depending on the species. For example, *M. kansasii* possessed most pathogenicity genes, whereas *M. gordonae* lacked some pathogenicity genes such as the secretion systems ESX-1, −2, and −3. We showed that mlstverse using mlstdb.NTM had higher sensitivity than the other methods and correctly identified all 29 samples at the subspecies level.

### Detection sensitivity and specificity

**Figure 4.**
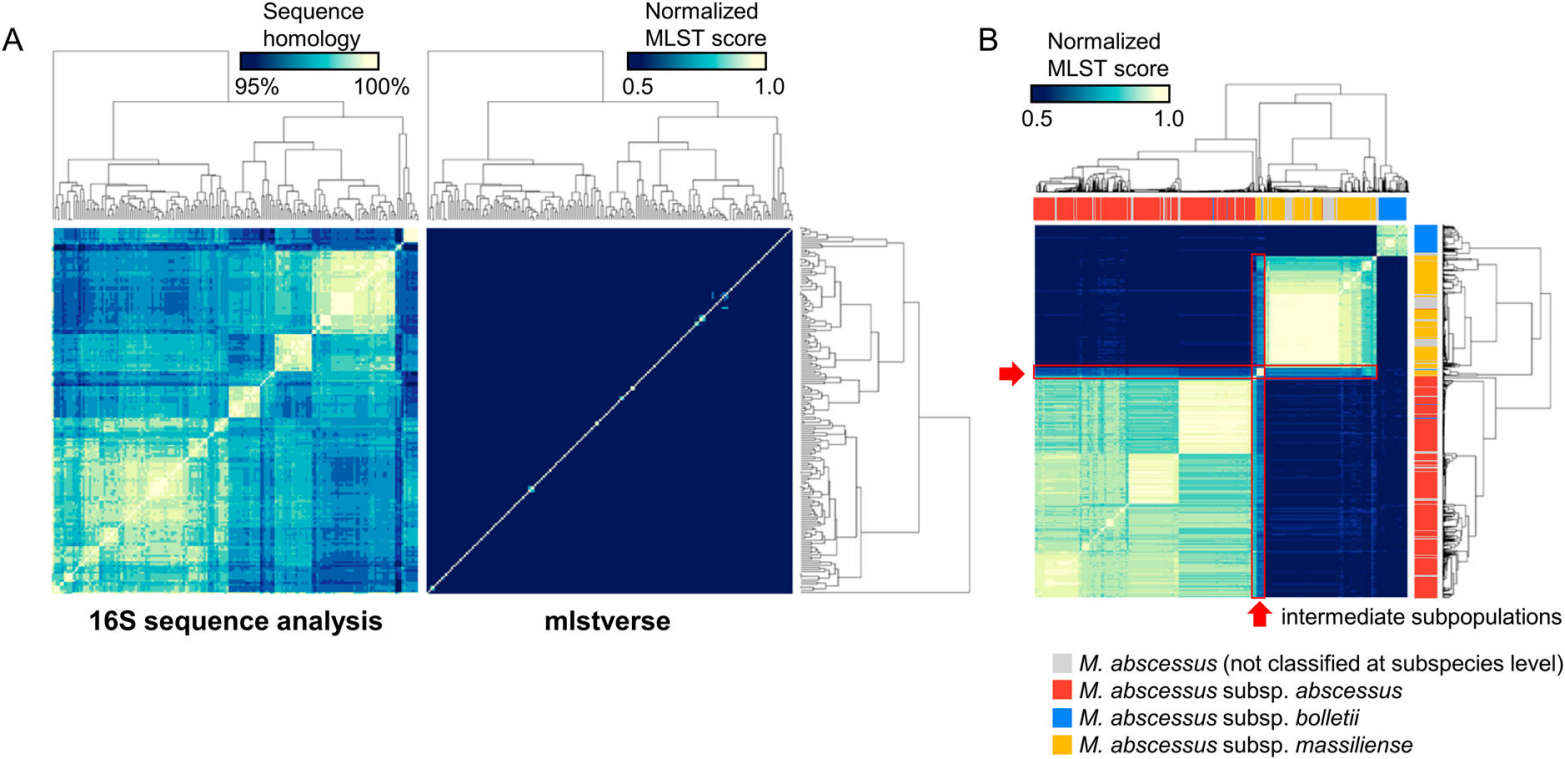
**Sensitivity and specificity.** A) Species-level evaluation of sequence homologies of 16S rRNA genes obtained from SILVA (left) and profile similarity in the mlstverse database (right). Species were ordered based on hierarchical-clustering results with complete-linkage. The profile shown on the right was sorted in the same order. B) Subspecies-level evaluation of *M. abscessus* profile similarities in the mlstverse database. The colour bar under the dendrogram indicates subspecies information stored in assembly metadata corresponding to subspecies that are not yet classified (grey), subsp. *abscessus* (red), subsp. *bolletii* (blue), or subsp. *massiliense* (yellow) (also see Figure S3).

We evaluated the sensitivity and specificity of mlstverse by identifying NTM species using all sequences in mlstdb.NTM as an input (Figure 4A). We calculated normalized MLST scores based on the cumulative sum of the mapping ratio for each gene, using mlstverse. Another identification method based on the sequence homology of the 16S rRNA genes was also performed for comparison purposes. Studying the sequence homology of the 16S rRNA genes identified only 11 of the 175 NTM species at the species level with over 98.7% identity. Moreover, none of the species within the *Mycobacteroides*, *Mycolicibacillus*, and *Mycolicibacter* subgenera could be distinguished. However, mlstverse identified all 175 species with an MLST score difference of 0.98 (on average), with a minimum difference of 0.1. We assessed the resolution to discriminate the subspecies of *M. abscessus*, based on differences in the MLST scores. Figure 4B shows the correlations of the scores obtained by MLST using 184 genes among *M. abscessus* subspecies. Significant score differences were found in MLST scores between the profiles of all combinations of different subspecies (*p* < 10^−15^). Only the scores from *M. abscessus* samples with subspecies labels were used in this test. Subspecies could be distinguished from each other by an MLST score difference of 0.38, on average. We also found two subpopulations of intermediate characteristics between *M. abscessus* subsp. *abscessus* and *M. abscessus* subsp. *massiliense* (shown with a red frame, Figure 4B, Figure S3). These subpopulations showed similar MLST scores, even though they were genetically different from other subspecies. The MLST analysis using 184 genes demonstrated clear discrimination among NTM species and the new subpopulation of *M. abscessus*.

### Rapid detection of NTM species by nanopore sequencing

**Figure 5.**
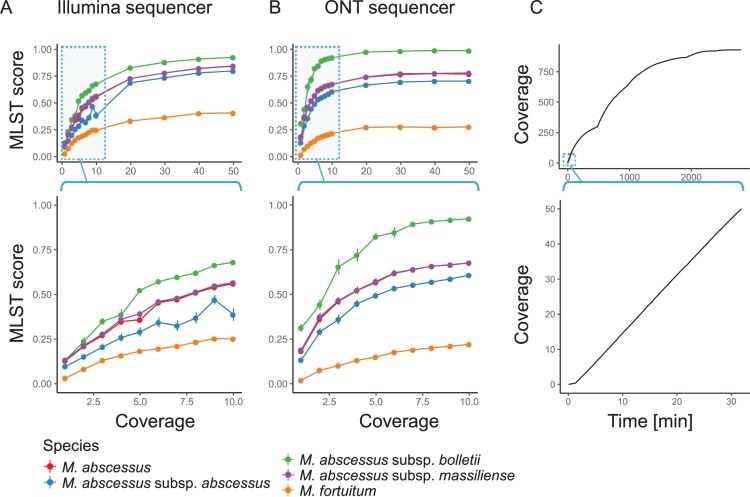
**Rapid detection of *M. abscessus* at the subspecies level.** A and B) MLST scores vs. coverage with Illumina short reads (A) and ONT long reads (B). Views based on a wide range of coverage (top of A and B) or focused on low coverage range (bottom of A and B) are shown. C) Amount of output data vs. time using an ONT MinION sequencer. Data obtained during the first 48 h (2,880 min; top) or initial 30 min (bottom) are shown.

Nanopore sequencing is the first NGS technology that enables the production of sequencing data in real time. Unlike previous NGS technologies, usable data can be obtained without waiting until the end of a sequencing run. Thus, if the amount of required sequencing data is very small, it is possible to rapidly identify the NTM species. The additional benefit of a small amount of required data is the ability to handle many clinical samples in parallel on a sequencing run. To estimate the minimum amount of data required for such identification, we examined two data sets of *M. abscessus* subsp. *bolletii* obtained from Illumina and ONT sequencers, and then randomly extracted a subset of those data. The analyzed data of the extracted subsets showed that the MLST score increased with the amount of data used for identification, both with the Illumina (Figure 5A) and ONT (Figure 5B) platforms. It was also observed that the score difference between the target, *M. abscessus* subsp. *bolletii*, and other species expanded with an increased amount of sequencing data. We found that 10X long reads obtained from the ONT MinION or 30X short reads obtained from the Illumina HiSeq2500 were sufficient to saturate the MLST score. Considering the average 5 Mb-sized *Mycobacterium* genomes, we could, in theory, identify 100 samples per run using an Illumina MiSeq instrument (with 600 cycles) or 200 samples per run using the ONT MinION. For the rapid detection of NTM species, these results indicated that only 10 min of sequencing with the ONT MinION was sufficient to obtain an MLST score enabling identification (Figure 5C).

## Discussion

In this study, we expanded the number of NTM species with known genomic sequences to 175 and substantially improved the quality of genome assemblies. Comparative analysis using core and pan genomes constructed from 175 NTM genomes revealed the subgenera of the newly sequenced NTM species. This phylogenetic classification strongly reflected differences in the growth type that were consistent with previous studies [9,10]. However, we found several exceptions in the phylogenetic tree shown in Figure 1C. The first of these exceptions is in the subgenus classification of the phylogenetic tree. In the phylogenetic tree, several species such as *M. vulneris*, *M. doricum*, and *M. tusciae* showed different growth types compared to similar species in the same subgenus. Discrepancies in the growth type were also found by Gupta et al. (2018) and Fedrizzi et al. (2017), and they may be due to differences in several growth factors [9,10]. Among the newly classified subgenera of 27 species identified in this study, only one species (*M. bourgelatii*) showed a discrepancy in the growth type where the growth type was rapid, but it was classified among a slow-growth subgenus of *Mycobacterium*. According to the classification based on three CSIs and four CSPs, which are characteristic features specifically for the *Mycobacterium* subgenera (Figure S4), two of three CSIs and three of four CSPs were found in *M. bourgelatii*. Thus, it seems reasonable that *M. bourgelatii* was classified as a *Mycobacterium* subgenus.

We newly determined the genome of *M. terrae* ATCC 19619, which was originally found in 1967 as a different species called *M. novum*. In 1974, Meissner et al. reported that *M. novum* was synonymous to *M. terrae* [37]. In Meissner’s study at that time, they used phenotypic information with a binary value rather than genetic information to classify *Mycobacterium* species. After that, the genomic sequence of *M. terrae* ATCC 19619 was never determined to our knowledge. Using the genome data of *M. terrae* ATCC 19619 in this study, we compared this species with similar species of *M. terrae* CIP 104321, *M. terrae* NCTC 10856, *M. nonchromogenicum* DSM 44164, and *M. triviale* DSM 44153 (Figure S5). The genetic difference between *M. novum* and *M. terrae* was small compared to that between other species, but it was much larger than that between *M. terrae* strains. In the same analysis including 175 NTM species, several species were found to be closer to *M. terrae* than *M. novum* (Figure 1C). These results indicate that *M. novum* is likely a different species than *M. terrae*.

The taxonomic classification of the *M. abscessus* subspecies was reviewed several times, based on recent genome sequencing and MS studies. One important difference from a clinical viewpoint is resistance to macrolide antibiotics between *M. abscessus* subsp. *abscessus* and *M. abscessus* subsp. *massiliense*, which is characterized by the *erm(41)* gene [38–40]. The *erm(41)* gene is a functionally inducible erythromycin ribosome methyltransferase that confers resistance to macrolide antibiotics. Subspecies belonging to *M. abscessus* subsp. *abscessus* possess *erm(41)*, whereas those belonging to *M. abscessus* subsp. *massiliense* do not. The mlstverse software package enabled clear discernment of those major subspecies populations (*abscessus* and *massiliense*), even though *erm(41)* was not included in the MLST database. In addition, we discovered two intermediate subpopulations consisting of 43 strains between the *abscessus* and *massiliense* subspecies (Figure 4B, Figure S3), and these strains showed closely similar MLST scores against each *abscessus* and *massiliense* subspecies member. Conventional methods such as MS, 16S rRNA sequencing, and previous MLST-based analysis did not distinguish those major subspecies or find such subpopulations. Several guidelines recommend a different antibiotic regimen, depending on the observation of macrolide resistance [12,41], which is directly related to different subspecies of *M. abscessus*. An MLST-based identification with high resolution at the subspecies level could contribute to appropriate drug regimens for treating NTM diseases.

Currently, MS is being used in large hospitals and offers advantages in terms of high-speed detection and a low running cost. However, it was reported that rare species could not be detected [42,43], and that closely related species such as *M. chimaera* and other MAC species could not be distinguished correctly [44,45]. In fact, the MS analysis of 29 clinical samples in this study also showed misidentification of several species such as *M. gordonae* and *M. wolinskyi*. However, MLST-based identification using NGS showed higher accuracy and the potential to rapidly detect pathogens. Given the required amount of data for MLST-based identification, highly multiplexed sequencing could reduce the running cost of NTM identification to a level comparable with MS analysis.

Another possibility for sequencing-based detection is metagenomic shotgun analysis directly using clinical specimens without culture isolation. A limit of this study is that our method still requires cultured NTM cells similar to MS and other MLST-based detection methods and was not evaluated using metagenomic data. Recent studies demonstrated the direct detection of *M. tuberculosis* from clinical specimens of patients with respiratory diseases [46–48]. Because metagenomic data derived from clinical specimens generally contain a majority of the human genome and a limited number of pathogen sequences, vast amounts of sequencing data are required to detect the target pathogen. The method described here can potentially enable identification of NTM species using low-coverage data and, therefore, might be able to work with a portion of the target sequences available in metagenomic shotgun-sequencing data. Future applications of this methodology to genomic data from clinical isolates and metagenomic data from clinical specimens could improve disease epidemiology and increase the cure rates associated with NTM diseases.

## Supplementary Material

Supplemental Material

## Data Availability

Package and source code of mlstverse were available at GitHub (https://github.com/ymatsumoto/mlstverse). The BioProject identification number is PRJDB7717. The BioSample identification numbers are SAMD00153162 to SAMD00153224.
